# Effect of tranexamic acid irrigation on perioperative blood loss during mini‐percutaneous nephrolithotomy: A pilot double‐blind randomised controlled trial

**DOI:** 10.1002/bco2.70157

**Published:** 2026-01-20

**Authors:** Ornnicha Prohsoontorn, Kun Sirisopana, Surawach Piyawannarat, Yada Phengsalae, Premsant Sangkum, Wisoot Kongchareonsombat, Chinnakhet Ketsuwan

**Affiliations:** ^1^ Division of Urology, Department of Surgery, Faculty of Medicine Ramathibodi Hospital Mahidol University Bangkok Thailand

**Keywords:** blood loss, irrigation, mini‐PCNL, topical administration, tranexamic acid

## Abstract

**Objective:**

To evaluate the efficacy and safety of adding 0.1% tranexamic acid (TXA) to irrigation fluid in reducing perioperative blood loss during mini‐percutaneous nephrolithotomy (mini‐PCNL).

**Patients and Methods:**

In this prospective, randomised study, 40 patients undergoing mini‐PCNL were allocated to receive irrigation fluid containing either 0.1% TXA (*n* = 20) or distilled water (placebo; *n* = 20). The outcomes assessed included changes in haemoglobin, estimated blood loss, operative duration, irrigation volume, length of hospital stay, transfusion requirements, stone clearance and TXA‐related adverse events.

**Results:**

Baseline characteristics were comparable between the two groups. The TXA group had significantly less haemoglobin decline (0.5 g/dl vs. 1.5 g/dl) and lower estimated blood loss (91.7 ml vs. 169.0 ml) compared with the placebo group (both *p* < 0.05). Operative time and hospital stay were also shorter in the TXA group (*p* < 0.05). Transfusion rate and irrigation volume were lower in the TXA group, while stone clearance rates were comparable between the groups (90% vs. 85%; *p* = 0.633). No TXA‐related adverse events were observed.

**Conclusion:**

The addition of 0.1% TXA to irrigation fluid during mini‐PCNL significantly reduces perioperative blood loss and appears to be safe in this pilot cohort, without increasing complications observed in the study.

## 1 INTRODUCTION

Percutaneous nephrolithotomy (PCNL) is widely recognised as the gold standard for the management of large renal calculi (>2.0 cm), as recommended by the European Association of Urology^1^ and the American Urological Association guidelines.^2^ In comparison with other minimally invasive modalities, including shock wave lithotripsy (SWL) and retrograde intrarenal surgery (RIRS), PCNL consistently demonstrates superior stone‐free rates.^1^, ^2^ Nevertheless, conventional PCNL is associated with considerable morbidity, particularly haemorrhagic complications,^3^, ^4^ which has prompted the development of less invasive yet equally efficacious alternatives.

Mini‐PCNL, initially introduced by Jackman et al.^5^ for paediatric applications, has subsequently gained broad acceptance in adult practice because of its reduced tract size and lower procedural invasiveness. This technique has been associated with decreased intraoperative complication rates, reduced postoperative pain and shorter hospitalisations.^6^

Integrating adjunctive pharmaceutical modalities into surgical procedures offers a simple yet effective way to enhance surgical efficiency and reduce the risk of serious complications. Tranexamic acid (TXA), an antifibrinolytic agent, has been widely utilised across a range of surgical settings—including endoscopic sinus surgery, knee arthroplasty, dental surgery, caesarean section, open‐heart surgery and liver transplantation^7^, ^8^, ^9^, ^10^, ^11^, ^12^—as well as in urological procedures, such as radical prostatectomy, transurethral resection of the prostate, and conventional PCNL,^13^, ^14^, ^15^ to effectively reduce intraoperative blood loss. Despite its well‐established efficacy, the use of TXA as an additive to irrigation fluid during mini‐PCNL has not been systematically evaluated.

## 2 PATIENTS AND METHODS

This study was designed as a double‐blind, placebo‐controlled, prospective, randomised trial conducted at the Faculty of Medicine, Ramathibodi Hospital, Thailand, between July 2020 and February 2021. The study was carried out in compliance with the 2010 Consolidated Standards of Reporting Trials (CONSORT) guidelines. Ethical approval was obtained from the Institutional Review Board (COA.MURA2020/579), and the trial was registered with the Thai Clinical Trials Registry (TCTR20200612002). Written informed consent was obtained from all participants prior to randomisation. Patients diagnosed with renal calculi and scheduled for mini‐PCNL were eligible for inclusion. Patients were excluded if they had TXA hypersensitivity or defective colour vision; were receiving anticoagulant therapy; or had a history of unstable cardiovascular disease, subarachnoid haemorrhage, abnormal liver function, acute or chronic renal failure, or haematological disorders.

**FIGURE 1:**
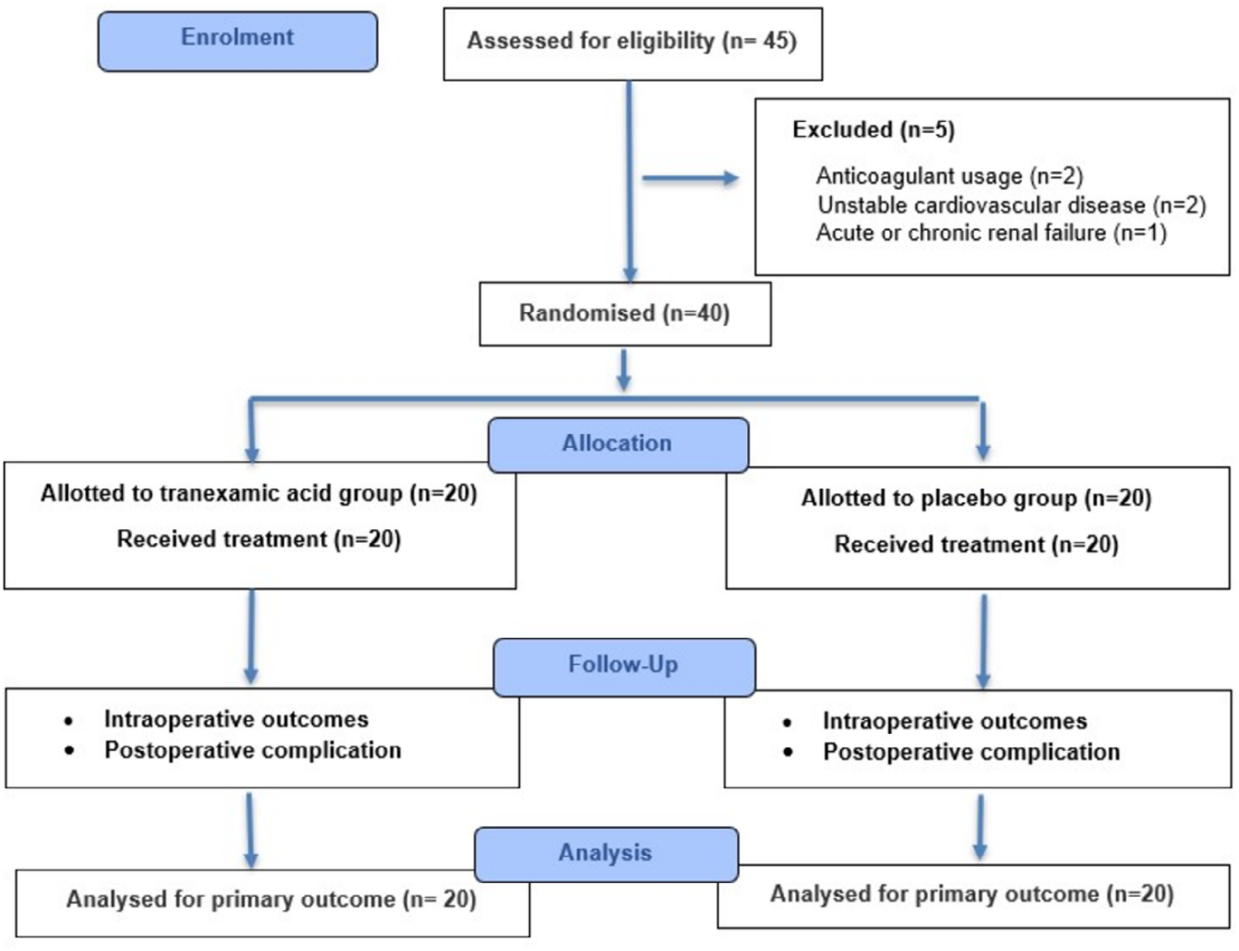
Flow of patients during the study.

Eligible patients were randomised in a 1:1 ratio using computer‐generated permuted blocks, with allocation concealed in sequentially numbered, sealed, opaque envelopes and assigned to one of two groups (Figure 1 consort flow diagram). The TXA group received a 0.1% tranexamic acid solution, which was prepared by diluting 1000 mg (10 ml) of TXA in 1 L of normal saline irrigant. The placebo group received a solution prepared by diluting 10 ml of distilled water in 1 l of normal saline irrigant. The consort checklist for the article has been added in the Supporting Information.

All procedures were performed by a single surgeon (CK), who was blinded to group allocation. Both groups followed an identical standardised surgical protocol. The patients were positioned in the Galdakao‐modified supine Valdivia (GMSV) position. A 5 Fr ureteric catheter was inserted cystoscopically, and retrograde pyelography was performed. Percutaneous renal access was achieved using the triangulation technique under fluoroscopic guidance with an 18‐gauge needle. Tract dilation was carried out over a guidewire, and a 16 Fr nephrostomy sheath was inserted. Stone fragmentation was performed using a 12 Fr nephroscope (MIP‐M system, Karl Storz, Tuttlingen, Germany) and a 120 W Ho:YAG laser (Lumenis, San Jose, CA, USA) with a 550 μm fibre (1.5 J, 30 Hz). At the conclusion of the procedure, a 6 Fr double‐J ureteral stent was placed in all patients. The stent was removed 2 weeks post‐operatively. Residual stones were evaluated by non‐contrast computed tomography of the kidneys, ureters and bladder (CT KUB) on post‐operative day 7.

Baseline demographic and stone characteristics were documented pre‐operatively. Intra‐operative and post‐operative data were prospectively collected, including haemoglobin and haematocrit changes, estimated blood loss, need for blood transfusion, total irrigation volume, operative duration, hospital length of stay, perioperative complications and adverse events associated with TXA. Stone‐free status was defined as the absence of residual stones or the presence of fragments ≤4 mm, as confirmed by non‐contrast CT KUB on post‐operative day 7. At our institution, transfusions were indicated for haematocrit <30% or haemodynamic instability. All post‐operative assessments were performed by an independent investigator (OP) blinded to the treatment allocation.

### 2.1 Outcomes

The primary endpoint was to evaluate the efficacy of TXA in irrigation fluid for reducing perioperative blood loss during mini‐PCNL. This was assessed by measuring post‐operative haemoglobin and haematocrit decline, as well as total estimated blood loss. The secondary endpoint was to assess the effect of TXA on perioperative complication rates associated with mini‐PCNL.

### 2.2 Sample size

Based on a previous PCNL study,^16^ the minimum sample size was calculated using STATA software version 14.1 (StataCorp, College Station, TX, USA). The calculation indicated that at least 11 patients per group were required to detect a difference in mean fall in haematocrit (4.2 vs. 7.8) with standard deviations of 2.6 and 3.2, assuming a two‐sided alpha of 0.05 and a power of 80%. To accommodate potential dropouts, the calculated sample size was increased to a total of 40 participants.

### 2.3 Statistical analysis

The data were analysed using STATA version 14.2 (StataCorp, College Station, TX, USA). Quantitative variables are presented as mean ± standard deviation (SD), and categorical variables are presented as frequencies and percentages. Between‐group comparisons were performed using the independent *t*‐test for continuous variables and Fisher's exact test for categorical variables. A *p*‐value < 0.05 was considered statistically significant.

## 3 RESULTS

A total of 40 patients with renal calculi requiring mini‐PCNL were enrolled and randomised equally into the TXA group (*n* = 20) and the placebo group (*n* = 20) (Figure 1). The baseline demographic and clinical parameters were comparable between the groups (Table 1). The perioperative outcomes are presented in Table 2. The TXA group demonstrated a significantly smaller mean haemoglobin reduction compared with the placebo group (0.5 ± 0.3 vs. 1.5 ± 0.9 g/dl, *p* = 0.014), as well as lower estimated blood loss (91.7 ± 69.4 vs. 169.0 ± 145.5 ml, *p* = 0.041). The mean operative time was significantly shorter in the TXA group (96.5 ± 23.7 vs. 123.5 ± 22.7 min, *p* < 0.001). Although the TXA group required a lower volume of irrigation fluid, the difference was not statistically significant (7.1 ± 2.5 vs. 9.2 ± 4.2 l, *p* = 0.066). The mean length of hospital stay was significantly reduced in the TXA group (2.7 ± 0.8 vs. 3.5 ± 1.1 days, *p* = 0.008). Complete stone clearance was achieved in 90% of TXA cases and 85% of placebo cases (*p* = 0.633). No patient in the TXA group required a blood transfusion, compared with one patient (5%) in the placebo group (*p* = 0.311). Post‐operative complications occurred in 25% (5/20) of TXA patients and 30% (6/20) of placebo patients (*p* = 0.744), with severity classified according to the Clavien–Dindo system (Table 3). No patient required angioembolisation. Importantly, no adverse events were attributable to TXA administration in the irrigation fluid.

**TABLE 1:** Patient demographics and preoperative stone characteristics.

Patient characteristics	Tranexamic acid group (*n* = 20)	Placebo group (*n* = 20)	*p*‐value
Age (years) (mean ± SD)	58.9 ± 7.7	61.2 ± 6.6	0.311
Gender: *n* (%)			
Male	7 (35.0)	7 (35.0)	1.000
Female	13 (65.0)	13 (65.0)	
Laterality			
Left	8 (40.0)	10 (50.0)	0.525
Right	12 (60.0)	10 (50.0)	
BMI (kg/m^2^) (mean ± SD)	25.9 ± 5.5	24.9 ± 5.4	0.295
History of previous interventions (*n*)	3	4	0.677
Type of previous intervention			
PCNL (*n*)	1	1	1.000
ESWL (*n*)	2	3	0.633
Diabetes mellitus: *n*	2	3	0.633
Hypertension: *n*	3	3	1.000
Dyslipidaemia: *n*	4	3	0.677
ASA classification: *n* (%)			
Class 1	16 (80.0)	17 (85.0)	0.526
Class 2	2 (10.0)	3 (15.0)	
Class 3	2 (10.0)	0 (0.0)	
Stone size (mm^2^) (mean ± SD)	422.3 ± 189.1	441.2 ± 175.7	0.746
Skin‐to‐stone distance (cm) (mean ± SD)	9.1 ± 0.8	8.7 ± 0.8	0.201
Stone density (Hounsfield units) (mean ± SD)	836.8 ± 297.7	841.8 ± 240.8	0.954
Grade of hydronephrosis: *n* (%)			
No or mild	16 (80.0)	16 (80.0)	1.000
Moderate or severe	4 (20.0)	4 (20.0)	
Number of accesses: *n* (%)			
Single	17 (85.0)	18 (90.0)	0.633
Multiple	3 (15.0)	2 (10.0)	
Calyx of puncture: *n*			
Lower	16	15	0.597
Middle	5	5	
Upper	2	2	
Preoperative eGFR (ml/min/1.73 m^2^) (mean ± SD)	87.6 ± 11.6	90.2 ± 10.6	0.457
Preoperative Hb (g/dl) (mean ± SD)	12.8 ± 1.5	12.7 ± 1.5	0.825
Preoperative Hct (%) (mean ± SD)	38.6 ± 4.7	37.3 ± 9.5	0.615

**Abbreviations:** ASA, American Society of Anesthesiologists; BMI, body mass index; eGFR, estimated glomerular filtration rate; ESWL, extracorporeal shock wave lithotripsy; Hb, haemoglobin; Hct, haematocrit; PCNL, percutaneous nephrolithotomy.

**TABLE 2:** Perioperative outcomes between the two groups.

Parameters	Tranexamic acid group (*n* = 20)	Placebo group (*n* = 20)	Mean difference (95% CI)	*p*‐value
Fall in haemoglobin (g/dl) (mean ± SD)	0.5 ± 0.3	1.5 ± 0.9	−1.00 (−1.42 to −0.58)	0.014*
Fall in haematocrit (%) (mean ± SD)	1.6 ± 0.8	4.3 ± 2.8	−2.70 (−3.98 to −1.42)	0.047*
Estimated blood loss (ml) (mean ± SD)	91.7 ± 69.4	169.0 ± 145.5	−77.30 (−147.95 to −6.65)	0.041*
Operative time (min) (mean ± SD)	96.5 ± 23.7	123.5 ± 22.7	−27.00 (−41.38 to −12.62)	<0.001*
Amount of irrigation fluid (l) (mean ± SD)	7.1 ± 2.5	9.2 ± 4.2	−2.10 (−4.24 to 0.04)	0.066
Blood transfusion requirement *n* (%)	0 (0.0%)	1 (5.0%)	‐	0.311
Length of hospital stay (days) (mean ± SD)	2.7 ± 0.8	3.5 ± 1.1	−0.80 (−1.40 to −0.20)	0.008*
Stone‐free status *n* (%)	18 (90.0%)	17 (85.0%)	–	0.633

* Statistically significant.

**TABLE 3:** Comparison of complications between groups classified using the Clavien–Dindo system.

Grade	Complications	Tranexamic acid group (*n* = 20)	Placebo group (*n* = 20)	*p*‐value
I	Fever	2	2	1.000
	Mild haematuria	2	1	0.548
II	UTI with fever leading to a change in antibiotics	1	2	0.548
	Haematuria requiring blood transfusion	0	1	0.311
	Total	5	6	

## 4 DISCUSSION

This randomised controlled trial demonstrates that the addition of TXA to irrigation fluid during mini‐PCNL significantly reduces perioperative blood loss, operative time and hospital stay without increasing the risk of adverse events. Specifically, the TXA group experienced a markedly smaller decline in haemoglobin and haematocrit, lower total blood loss, and shorter operative duration compared with the placebo group. These findings suggest that topical TXA administration via irrigation fluid represents a safe and effective strategy for optimising surgical outcomes in mini‐PCNL.

Bleeding remains a significant clinical concern in PCNL. Several factors have been associated with an increased risk of severe bleeding during PCNL, including diabetes mellitus, hypertension, urinary tract infection, renal anomalies, number and size of calculi, presence of staghorn stones, type of tract dilation, multiple punctures and larger tract sizes.^4^, ^17^, ^18^, ^19^, ^20^ The reported transfusion rates for conventional PCNL range from 3% to 23%.^21^, ^22^, ^23^ The aetiology of bleeding following percutaneous renal surgery most commonly involves injury to the renal parenchyma or vasculature. In most cases, such haemorrhage is self‐limiting and can be effectively managed with conservative measures, including temporary occlusion of the nephrostomy tube, application of local pressure at the access site and allowing the pelvicalyceal system to tamponade via clot formation. When bleeding is refractory to these measures, selective renal angioembolisation may be required. Although mini‐PCNL generally reduces transfusion risk,^24^, ^25^ our placebo group still demonstrated measurable haemoglobin decline, confirming that haemorrhage remains relevant even in small‐tract surgery. In our study, the estimation of blood loss based on haemoglobin and haematocrit decline may have been influenced by perioperative hydration and haemodilution, potentially leading to underestimation or overestimation of the true blood loss.

Given that prevention is preferable to reactive management, strategies aimed at minimising intra‐operative bleeding are of substantial clinical importance. In our trial, no patients in the TXA group required transfusion, compared with 5% in the placebo group, underscoring the potential role of TXA as an adjunctive haemostatic measure in mini‐PCNL. Our findings are consistent with prior studies demonstrating TXA's efficacy in reducing blood loss across a wide range of surgical specialties. In standard PCNL, most investigations have evaluated intravenous TXA administration and have consistently reported decreased intra‐operative bleeding and transfusion rates. For example, Kumar et al.^26^ first reported TXA as a safe and effective agent in PCNL, noting a significantly smaller mean haemoglobin drop in the TXA group (1.39 g/dl) compared with the placebo group (2.31 g/dl; *p* < 0.0001). The authors concluded that TXA was well tolerated, reduced blood loss and lowered perioperative complications. Similarly, Meharwal et al.^15^ observed a haemoglobin decrease of 0.40 ± 0.24 g/dl and a PCV reduction of 1.81 ± 3.10 (*p* = 0.001) in patients receiving TXA.

Although systemic TXA administration is effective, it carries a theoretical risk of thromboembolic events, particularly in high‐risk patients. The topical route, as used in this study, offers the advantage of delivering high local drug concentrations at the surgical site while minimising systemic absorption and associated risks. The efficacy of topical TXA is well established across multiple surgical specialties. A Cochrane review by Ker et al.,^27^ involving 29 randomised trials, demonstrated significant reductions in transfusion rates and perioperative blood loss without an increase in thromboembolic complications. In spine surgery, Farzanegan et al.^28^ reported reduced bleeding with topical TXA during posterior laminectomy without higher complication rates. Comparable benefits have also been reported in orthopaedic, cardiac and plastic surgery. In standard PCNL, Bansal and Arora^16^ demonstrated that 0.1% topical TXA significantly decreased perioperative blood loss compared with placebo (154.5 ml vs. 212.6 ml, *p* < 0.001), along with a smaller haemoglobin drop (1.71 g/dl vs. 2.67 g/dl, *p* < 0.001).

Another notable finding of our study was the shorter operative time in the TXA group, likely attributable to improved intra‐operative visualisation from reduced bleeding, which may have facilitated more efficient stone fragmentation and retrieval. In our study, the TXA group also demonstrated a higher stone‐free rate, although the difference was not statistically significant. The overall complication rate in our trial was low and comparable between the groups, with no TXA‐related adverse events. This observation is consistent with previous findings,^16^ supporting the favourable safety profile of topical TXA when used in the irrigation fluid.

A major strength of this double‐blind randomised controlled trial is the use of allocation concealment, which minimises selection bias. All procedures were performed by a single experienced surgeon, ensuring procedural consistency. However, this study has several limitations. The relatively small sample size may have limited the ability to detect differences in rare outcomes and uncommon complications, indicating that the study was underpowered for these endpoints. Systemic TXA absorption was not measured, precluding pharmacokinetic assessment. Finally, the single‐centre setting in a high‐volume tertiary hospital may limit generalisability.

## 5 CONCLUSION

To our knowledge, this is the first pilot randomised controlled trial to evaluate topical TXA delivered via irrigation during mini‐PCNL. The addition of 0.1% TXA to irrigation fluid appeared to reduce perioperative blood loss, operative time and hospital stay without increasing complication rates. These findings provide preliminary, hypothesis‐generating evidence supporting the potential role of topical TXA as a practical adjunct to improve surgical outcomes in mini‐PCNL.

## AUTHOR CONTRIBUTIONS

**Ornnicha Prohsoontorn:** Conceptualisation; methodology; data curation; investigation; writing—original draft; writing—review and editing. **Kun Sirisopana:** Investigation; data interpretation. **Surawach Piyawannarat:** Investigation; data interpretation. **Yada Phengsalae:** Formal analysis; data interpretation. **Premsant Sangkum:** Data interpretation; validation; visualisation; writing—review and editing. **Wisoot Kongchareonsombat:** Investigation; data interpretation. **Chinnakhet Ketsuwan:** Project administration; data interpretation; validation; visualisation; supervision; writing—review and editing.

## CONFLICT OF INTEREST STATEMENT

The authors declare that they have no competing interests.
